# Case Report: Diagnostic itinerary of a male case of juvenile-onset systemic lupus erythematosus in Bouake

**DOI:** 10.3389/fped.2024.1426246

**Published:** 2025-02-04

**Authors:** Aissata Doucoure Traore, Joseph Kan Enock Koffi, Christ Ziahy Reine Marie Koffi, Konan Joe Clovis Yao, Jean Jacque Goua, Ehaulier Soh Christian Kouakou, Weu Melanie Tia, Felix Jean Claude Daboiko

**Affiliations:** ^1^ Department of Rheumatology, CHU Bouaké, Université Alassane Ouattara, Bouaké, Côte d'Ivoire; ^2^ Department of Nephrology, CHU Bouaké, UFR des Sciences Médicales de Bouaké (Service de Rhumatologie CHU Bouaké), Bouaké, Côte d'Ivoire

**Keywords:** JSLE, boy, late diagnosis, Bouaké, itinerary

## Abstract

Juvenile-onset systemic lupus erythematosus (JLES) is an autoimmune disease of unknown aetiology. It is more common in girls but can occur in boys. Its onset at an early age is more severe, causing potentially fatal damage if not treated early. It is a polymorphous condition, misleading at first and little known in our African populations. We report a case of 12 years of misdiagnosis in a 17-year-old boy living in a semi-rural environment. This case illustrates the shortage of paediatric rheumatologists and marks a breaking point in the diagnosis of the disease, which is sometimes difficult even for practitioners, making it difficult for sick children to access care. It is important to make the general public, as well as nurses and doctors, aware of the importance of early diagnosis for effective and efficient treatment.

## Introduction

Juvenile-onset systemic lupus erythematosus (JSLE) is a rare autoimmune disease of children. Only 10%–15% of cases are diagnosed before the age of 16. It is a polymorphous, multifactorial disease of unknown aetiology, more serious than in adults because of associated renal damage, and the cause of significant morbidity and mortality (1). Its onset at an early age is more severe and should prompt a search for an associated genetic abnormality. The worldwide prevalence of JSLE is estimated at between 1.89 and 25.7 per 100,000 children (2). In Africa, JSLE remains poorly documented, particularly in Côte d'Ivoire, with a reported hospital incidence of 1 case in 7 years in Abidjan (3). It is more common in girls, but can also affect boys. According to the Single Hub and Access point for paediatric Rheumatology in Europe (SHARE) recommendations, anti-nuclear antibody (ANA) positivity associated with at least two Systemic Lupus International Collaborating Clinics (SLICC) clinical criteria justifies the advice and referral of the patient to a paediatric rheumatologist (4). Like the rest of Africa, Bouaké is a semi-rural town where access to healthcare is a real challenge, due to a lack of interest and of practitioners specialising in the field (5). Diagnosis and treatment of JSLE must be early in order to avoid potential damage. We report the case of a 17-year-old boy who was diagnosed with JSLE over a period of 12 years.

## Patient and observation

This is a 17-year-old adolescent from a non-consanguineous marriage, born of a normal full-term pregnancy and an euto-cic delivery. He lived with his divorced mother, a shopkeeper in a semi-rural area. She had no history of autoimmunity. His childhood growth was normal. He had a long history of inflammatory arthralgias of the small joints of the hands and feet, which developed in a febrile context and were first reported in childhood.

The onset of joint flare-ups occurred at the age of 5, with partial remission in a context of recurrent non-evanescent fever until the age of 8. During this initial period, he was regularly seen and treated for malaria by nurses in first-contact health facilities (ESPC). When his arthralgias persisted, he was referred to a second-contact centre (general hospital). He was first seen by a general practitioner and then a paediatrician, who both suggested a vaso-oclusive crisis linked to sickle cell disease and rheumatic fever. Haemoglobin electrophoresis and throat swabs were normal. He was treated with non-steroidal anti-inflammatory drugs (NSAIDs), which partially improved his condition. This treatment was continued by the mother as a self-medication combined with traditherapy.

The second period in the course of the disease was marked by the onset of systemic damage. At the age of 10, the mother described a difficult, emotionally labile child with intermittent schooling due to attention and memory deficits, which increased with the parents' divorce. At the age of 13, he presented with small dyschromic malar spots of limited extent, which his parents thought were related to puberty acne, and was treated with self-medication with dermocorticoids. At the age of 16, he developed a persistent cough and fever. The mother consulted an anti-tuberculosis centre on the family's instructions. He was seen by a general practitioner and investigations revealed interstitial pneumonitis on chest x-ray and acid-fast bacilli (AFB) on sputum examination. He was treated for pulmonary tuberculosis for 6 months and declared cured.

At the age of 17, after 12 years, the skin and mucous membrane lesions had spread (photosensitivity with diffuse purpuric lesions without necrosis on the trunk and lower limbs, alopecia and new-onset mouth ulcers), the joints were affected with synovitis of the large joints, the general condition had deteriorated with an almost constant fever of 38°7C, he had been suffering from headaches for 2 months and his face was puffy in the morning. He underwent a rheumatology consultation and was admitted to hospital. Biological findings included a ESR of 150 mm in the first hour, CRP 37 mg/L, haemoglobin 7.3 g∕dl, lymphopenia, creatinemia 16 mg/L and proteinuria 3.6 g∕24 h. Imaging demonstrated polyseritis (left pleurisy, ascites lamina and pericarditis). On immunology, ANA (1,280 speckled aspect), Anti-native DNA (>15 IU/ml), Anti-Sm (142 U/ml), aCL (15 U MPL/ml), U1RNP (135 U/ml) were positive. The diagnosis of JSLE was based on the EULAR-ACR 2019 criteria (38 points) and SLICC (11 criteria). A renal biopsy showed histological evidence of stage V lupus glomerulonephritis. The disease was at the first visit severe, with a SLEDAI index of 37. As part of a multidisciplinary approach (rheumatologist, internist, nephrologist, ophthalmologist, psychiatrist, haematologist and paediatrician), he was treated with high-dose bolus corticosteroids (Prednisone 60 mg/m^2^ per day) combined with adjuvant measures, followed by oral corticosteroids at 2 mg/kg per day and then tapering off as recommended. A visit was made to the ophthalmologist before starting hydroxychloroquine (400 mg/day) combined with cyclophosphamide (150 mg/day). The aim of the treatment and short- and medium-term follow-up was to control the relapse, preserve vital functions and achieve remission while preserving the child's schooling. Therapeutic education is an important part of treatment. In order to reduce the deleterious effects of the medication, the child should be monitored first on a weekly basis for 1 month, with blood glucose, blood count, creatinemia and serum protein electrophoresis checks; then on a monthly basis for 3 months and then every 3 months. Background treatment will be re-evaluated at 3 months and adapted for early cortisone sparing at a dose of less than 0.3 mg/kg/day. The clinical course was favourable after 4 weeks of treatment, with regression of the biological inflammatory syndrome. However, the severe renal, haematological and neuropsychological damage, which was discovered late, suggests a poor prognosis for the adolescent.

## Discussion

Our aim in this case study was to emphasise the need for information, training for practitioners, to lack of disease recognition and low referal in order to promote access to care for children with rheumatic diseases in Africa, and particularly in Côte d'Ivoire. JSLE is a extermely rare disease in Côte d'Ivoire, with only one case reported in Abidjan in 7 years, because largely underdiagnosed (3). The sex ratio of girls to boys with JSLE is lower than in adults, ranging from 1:5 to 1:18 (6, 7). In general, it is more severe in males, with more frequent renal failure, haematological disease, and neurological and cardiovascular impairment (8). Our patient was male. Our patient had no growth disorders or development of sexual characteristics. This is in contrast to the 8%–16% of JSLE described in the literature (9).

Symptoms, consisting of inflammatory arthralgia and intermittent fever with progressive onset, began at the age of 5 years. JSLE generally has an insidious and progressive onset, with variable and non-specific initial symptoms such as asthenia and fever, as was the case in our patient (10, 11). During this initial period (5–8 years), the mother had consulted nurses, a general practitioner and a paediatrician without success. The diagnoses mentioned were malaria, sickle cell disease and AAR. Paediatric health issues in sub-Saharan Africa are dominated by infectious diseases, in particular malaria, HIV/AIDS, nutritional disorders (malnutrition) and neonatal deaths (12, 13). Paediatric practice has naturally developed along these lines, with little interest in less frequent diseases (14), such as rheumatological conditions. In the West, however, paediatric rheumatology services are becoming increasingly important (15). The Ivorian healthcare system is pyramid-shaped, with first contact healthcare establishments (ESPC) at primary level. The lack of qualified staff at this level could be a factor in the long delay in diagnosing the disease.

The second period marked the onset of systemic damage at the age of 10, with intermittent treatments such as prolonged self-medication at the whim and despair of the mother. The problem of self-medication remains a topical one throughout the world. In Africa, the problem of self-medication concerns vulnerable populations. In the DRC, a study showed that 59.6% of patients were self-medicated before admission, and that this was the first recourse of mothers in cases of fever in children (16). This is thought to be linked to poverty and the proliferation of the illicit parallel market (17). The patient's mother was a small-scale market trader on a low income, living in a semi-rural area. Our patient had been self-medicating with anti-inflammatory drugs and traditherapy since childhood. Our patient had experienced difficulties at school due to attention and memory deficits, which had worsened with the divorce of his parents. At the age of 10, he was in elementary school. It has been noted that 50% of children and adolescents with lupus develop neuropsychological disorders, including 5%–10% with psychosis (18). There seems to be a causal relationship between father-mother conflicts and the mental state of children in general, and in particular those suffering from inflammatory diseases such as JSLE (19, 20). Mucocutaneous manifestations occur in 3/4 of cases, atypical, discreet; unlike adult lupus with the classic butterfly wing or vespertilio rash (10). At the age of 13, our patient presented with non-specific dyschromic lesions on the face, and at the age of 17 on the trunk and lower limbs. SLE is particularly misleading in adolescence. The disease may affect a single organ, but the systemic form is the usual form of manifestation, as in the case of our patient (11, 21). The patient had polyarteritis and a history of pulmonary tuberculosis. Articular manifestations affect nearly 80% of cases in children (1, 10). The patient had a long history of inflammatory arthralgia of the small and large joints since the age of 5. The patient described intermittent headaches of varying intensity, sometimes with insomnia. In children, headaches can resemble migraines, but can also be a symptom of serious complications such as meningitis, vasculitis or even cerebral thrombosis. Central nervous system involvement is a major cause of morbidity and mortality in children with SLE (22). When the disease was diagnosed, in addition to joint and skin involvement and neuropsychological disorders, there were haematological manifestations such as inflammatory anaemia, lymphopenia and renal manifestations marked by WHO stage V lupus nephropathy. Inflammatory anaemia affects 70% of lupus children and is a major factor in their severity (23, 24). Opportunistic infections are responsible for a large proportion of the morbidity and mortality in JSLE and are related to lymphopenia, as in our patient who presented with pulmonary tuberculosis at the age of 16. In contrast to adults, JSLE is more severe because of renal involvement (25, 26). The literature describes that 75% of affected children develop lupus nephropathy (1). Arthritis, rash, fever and renal involvement are the most common features of the paediatric form (10, 27), as observed in our patient.

Inflammatory and immunological tests [ANA, anti-native DNA, anti-Sm, anti-RNP, anti-cardiolipin antibodies (aCL)] were positive in our patient. These autoantibodies are more frequent in JSLE, particularly anti-Sm and anti-RNP antibodies (26). Almost half of children with SLE have aCL or a lupus-type circulating anticoagulant, as was the case in our patient (28). Only a small number develop thrombotic disease (28, 29). JSLE is more severe than the adult form (mean SLEDAI 10.2), which is confirmed in our patient (SLEDAI 37) (22, 30). The literature describes a higher prevalence of progressive and severe forms in patients of African and Asian origin (1). Treatment follows the same recommendations as for adults (1, 7). It combines high-dose corticosteroid therapy, hydroxychloroquine and immunosuppressants, biotherapy being unavailable due to its high cost. Corticosteroid therapy, which is often necessary in children, should be used early and in high doses in severe forms, in accordance with recommendations (4). Hydroxychloroquine should be prescribed systematically, as in the case of our patient. The diagnosis of the disease is made at the stage of complications. The management is multidisciplinary, well codified, centred on the treatment of these complications. psychosocial follow-up of the adolescent and also of the mother is necessary. in the light of the recommendations. The main obstacle to follow-up is the mother's poverty, which could be improved by equitable access to care.

## Conclusion

Over and above the 12 years of diagnostic wandering, there is a breaking point in the diagnosis and optimal management of the disease, which is sometimes difficult even for practitioners. The lack of a paediatric rheumatology unit and misinformation among the general public, which encourages self-medication and traditional therapies, are just some of the reasons why access to treatment is so difficult. It is important not only to raise public awareness, but also to train nurses and doctors in the importance of early diagnosis for efficient and effective treatment, in order to avoid the potential damage caused by JSLE. Children's health and well-being are inextricably linked to the physical, emotional and social health of their parents, social circumstances and children's educational practices.

## Data Availability

The original contributions presented in the study are included in the article/Supplementary Material, further inquiries can be directed to the corresponding author.
